# Design and feasibility of an implementation strategy to address Chagas guidelines engagement focused on attending women of childbearing age and children at the primary healthcare level in Argentina: a pilot study

**DOI:** 10.1186/s12875-022-01886-6

**Published:** 2022-11-08

**Authors:** Karen Klein, Javier Roberti, Mariel Rouvier, Maria Belizan, Maria Luisa Cafferata, Amanda Mabel Berrueta, Juan Pedro Alonso

**Affiliations:** 1 Institute of Clinical Effectiveness and Health Policy (IECS), City of Buenos Aires, Argentina; 2 National Scientific and Technical Research Council (CONICET), City of Buenos Aires, Argentina; 3 Ministry of Public Health of Chaco, Resistencia, Chaco, Argentina

**Keywords:** Chagas, Primary health care, Implementation strategy, Feasibility, Guideline, Effective intervention

## Abstract

**Background:**

Chagas is a public health problem, especially in Latin America, exacerbated by migratory movements and increasing urbanization. Argentina is among the countries with the highest estimated prevalence in the region, with 1,500,000 people infected, with mother to child as the main mode of transmission. Vertical transmission has been significantly reduced by treating women of childbearing age; several guidelines in the region recommend treatment as a primary prevention strategy for the child and a secondary prevention strategy for women and their families. Despite recommendations, women of childbearing age are not always treated, and children do not receive timely diagnosis and treatment. The objective of this research was to design an implementation strategy to improve using Chagas guidelines focused on attending women of childbearing age and children at the primary healthcare level and pilot it in three primary health care centers in Argentina.

**Methods:**

We conducted a pilot feasibility study using the Consolidated Framework for Implementation Research. A qualitative process evaluation was conducted using semi-structured interviews with health care providers and observations in primary health care centers.

**Results:**

We developed a multifaceted implementation strategy including training, flowcharts and reminders, a register of suspected and confirmed cases, and the selection of a management facilitator. The pilot study took place between September 2019 and May 2020. The implementation level was heterogeneous and varied depending on the components, being the facilitating factors, the simplicity of the intervention, professionals’ willingness to expand the indication of serologic tests, and staff commitment to the adoption of intervention components. The main barriers found were the change of authorities at the local level, some professionals´ reluctance to administer etiological treatment, staff shortages, lack of diagnostic supplies, and the health emergency caused by the COVID-19 pandemic.

**Conclusions:**

Behavioral change strategies should be applied to improve implementation to address some of the main barriers, including support actions offered by opinion leaders, medical experts, and local health authorities. Rapid diagnostic tests should be readily available to maintain behavior changes. We suggest further refinement of the strategy and its implementation in more centers to assess outcomes prospectively with a hybrid implementation research design.

**Supplementary Information:**

The online version contains supplementary material available at 10.1186/s12875-022-01886-6.

## Background

Chagas is a public health problem worldwide, especially in Latin America [1], exacerbated by migratory movements and increasing urbanization [2, 3]. Argentina is among the countries with the highest estimated prevalence in the region, with 1,500,000 people infected,, over 370,000 people affected by heart disease of Chagasic origin [1], with mother to child as the main mode of transmission [4, 5]. Vertical transmission has been significantly reduced by treating women of childbearing age [6–8]; several guidelines in the region recommend treatment as a primary prevention strategy for the child and a secondary prevention strategy for women and their families [9, 10]. Treating children has also proven to be effective and safe [11, 12].

Despite recommendations, women of childbearing age are not always treated, and children are not diagnosed in time because of several barriers at different levels: lack of access to diagnosis tests, complex diagnosis and treatment processes, lack of a pediatric formulation, inadequate medical training, inaccessible specialized care, drug toxicity, insufficient patient and healthcare provider awareness, and shortages of medication [13–16]. Chagas disease is integrated into national primary care policy: the national Chagas guideline [9] recommends that patients with *T. cruzi* infection be managed at the primary healthcare level since this facilitates access to diagnosis, treatment, and follow-up. To promote healthcare providers’ adherence to national recommendations for a comprehensive approach to Chagas disease in Argentina [9], we designed a multi-component implementation strategy based on elements with proven effectiveness in behavior change [17–20]. This strategy was adapted after a formative research evaluation aimed at assessing stakeholders’ perspectives on the acceptability and feasibility of the proposed implementation [14].

The objective of this research was twofold: we designed an implementation strategy for the use of Chagas guidelines focused on attending women of childbearing age and children at the primary healthcare level, piloted it and assessed feasibility and factors that influenced implementation.

## Methods

### Study design

We conducted a pilot feasibility study based on the implementation research approach [21] and using the Consolidated Framework for Implementation Research (CFIR) [22]. The intervention was piloted for nine months in three primary health care centers (PHCs) in northern Argentina.

A process evaluation was performed using qualitative methods [23, 24], collecting data through semi-structured interviews (developed by the research team and not previously published, see Additional file 11) and observations at the PHCs. The findings are reported in accordance with relevant reporting guidelines [25], see Additional file 12.

### Setting

This study was performed in Resistencia, capital city of Chaco Province, Argentina. Resistencia is a metropolitan area with a population of approximately 400,000 inhabitants. The city receives a constant flow of migrant population from rural endemic areas in the province. The National Chagas Program categorizes Chaco as a high-risk area [4]. However, entomological surveillance of *T. cruzi* infection in dwelling, triatomines, and animals has been carried out in a sustained manner in Argentina and particularly in Chaco, for more than a decade, with a significant decrease in vectorial cases [26].

Resistencia has 31 PHCs and three hospitals where Chagas cases are referred to. Three PHCs process samples at their own laboratories, whereas the other PHCs send samples to a central laboratory in Chaco Province weekly or refer patients to this laboratory for testing. The Provincial Chagas Program, part of the Chaco Provincial Epidemiology Department, provides the etiological treatment of Chagas disease to all users of the public health system in the province. The program also registers and monitors all cases treated.

### Sites and population

PHCs were selected if they were in areas with interrupted vector transmission, had no Chagas-related active government projects or other research project that could interfere with the intervention, and had at least one professional defined as a direct user of the implementation strategy (pediatricians, general practitioners, family physicians, gynecologists or obstetricians). PHCs also had to serve a varied population, including, for example, indigenous people among their users. The final selection of centers was agreed upon with provincial and city health authorities. PHC teams participated in this study, including managers, general practitioners, family physicians, gynecologists, obstetricians, obstetrics, pediatricians, and nurses. Other professionals, such as community health agents and technical professionals, also worked in the PHC but were not included in the study.

### Description of the implementation strategy

We developed a multifaceted implementation strategy including training, the use of flowcharts and reminders, a register of suspected and confirmed Chagas cases, and the selection of a management facilitator in each PHC (see Table 1). The theoretical basis for the development of the strategy included a systematic review of effective strategies for implementing clinical practice guidelines [27], the Platform for the comprehensive care of adults with Chagas disease that improved access to diagnosis and treatment in Bolivia [17], and the Patient-Centered Care Model from Colombia [28]. In addition, the strategy was tailored and adapted through formative research [14].

**Table 1:** Components of the implementation strategy

Component	Description
**Training**	All health care providers working with the population of interest were trained on Chagas disease and resource management
**Flowcharts on Chagas management and approach**	Flowcharts in poster format that included information on risk screening (questions to detect the population to be tested), procedures to manage people at risk and cases of Chagas disease, under national guidelines. The posters were tailored to the type of patient: for pediatric population, for prenatal controls and for women of reproductive age (see Additional files 1, 2, 3, 4, 5 and 6)
**Reminders**	Labels as reminders and to easily locate medical records of infected or at risk people
	Posters to remind professionals to ask questions to verify the risk of Chagas disease (risk screening), diagnose, follow-up results and treat positive cases (see Additional files 7, 8, 9 and 10)
**Frequently asked questions for patient management and approach**	This document summarized information to optimize the use of resources for managing Chagas disease at the first level of care, regarding notification of positive cases, access to etiological treatment, and coordination with other levels of care.
**Identification of clinical records of people at risk and positive cases**	The medical records of patients at risk of Chagas disease were identified (e.g., brightly coloured labels) to facilitate tracking results of diagnostic tests. Positive serologic tests results were recorded to facilitate routine practice. Family contacts needed to be followed up. Each healthcare center could design their own registration strategy
**Management facilitator**	Central role in the dissemination and implementation of all components and liaison between key actors and levels of care, contributing to adequate healthcare

The training was directed to the health care providers in charge of the target population (pregnant women, children, adolescents, women of childbearing age). It covered the use of the national guidelines for the management of Chagas disease (early detection of cases and those living with them, diagnosis, treatment and follow-up, and resource utilization) [9]. The training comprised a four-hour, face-to-face session. Flowcharts were distributed among healthcare staff; distributed material included risk screening tools, a description of procedures for managing people at risk and cases of Chagas disease, and printed reminders to show diagnostic tests and follow-up cases (see Additional files 1, 2, 3, 4, 5, 6, 7, 8, 9 and 10). The team's researchers developed the design of the flowcharts and other support materials based on the national guidelines and adapted them according to the findings obtained in the formative research [14]. PHCs were encouraged to identify medical records of patients at risk and to register positive cases and their cohabitants. Posters and leaflets designed by the national Chagas program, were also made available to PHC users. Finally, an opinion leader (named the management facilitator) was appointed in each PHC to allocate resources and communicate with the research team. A frequently asked question sheet with information on the management of positive cases at the first level of care supported the role of opinion leaders.

### Outcomes: process evaluation

The implementation process evaluation was based on implementation outcomes [29] and the CFIR [22]. This framework describes a list of dimensions and constructs that influence implementation, such as intervention characteristics, context, external factors, and the people involved.

The implementation outcomes were evaluated through semi-structured interviews with health personnel and observations at the centers during and at the end of the intervention, as described in the data collection section. The implementation outcomes we evaluated were adoption, fidelity, acceptability, appropriateness, and feasibility, following Proctor et al.’s framework [29].

Adoption, defined as the effective utilization of the intervention components, was evaluated at the facility level. A component was considered fully adopted when the interviewed healthcare personnel stated that they had consistently implemented it at different times during the evaluation. Those components that only some informants (or none) mentioned having used were considered partial or not implemented. For some components, such as the use of flowcharts and posters, observations were also used, besides the interviews, to define adoption. Fidelity, the degree to which the component was implemented as it was intended by the research team, was assessed at the healthcare staff level through the interviews. We considered that a component was implemented with high fidelity when informants stated that they used it as intended, and low fidelity when interventions were used differently or unsystematically. Acceptability and appropriateness were assessed through the perceptions of healthcare providers as seen in the interviews. We defined acceptability as the perception among the healthcare professionals at centers that the intervention components were acceptable, appropriate and relevant. Feasibility, the perception that the intervention components could be successfully implemented at each health center, was also assessed based on the perceptions of the interviewees. Those components that the interviewees consistently considered possible to be implemented were considered feasible, and those that some informants considered difficult to implement as planned or that were not adopted at the health centers were considered to be of low feasibility.

### Data collection

Qualitative semi-structured interviews [24, 30] were triangulated with data collected from observations and telephone interviews.

The research team conducted monthly telephone interviews with facilitators at PHCs and collected information on the availability of materials. Additionally, a researcher visited each PHC twice during the study period, one month after the intervention started and halfway through the intervention. A planned third visit to each PHC had to be cancelled because of social isolation measures during the COVID-19 pandemic, and data collection was performed remotely. Twenty-two semi-structured interviews with PHC directors, facilitators, and healthcare staff were performed. A guide was developed to cover the acceptability, implementation of components, and barriers and facilitators. Additionally, during the visits, observations were made to register the use and availability of printed materials and the use of the recording systems. Data collected during these observations were recorded on specially designed forms.

### Data analysis

Data collected through telephone interviews and observation were entered into the Research Electronic Data Capture (REDCap) system. Interviews were recorded, transcribed verbatim, and entered into Atlas.ti v8 (Scientific Software Development GmbH, Berlin, Germany). Following familiarization with the dataset, a first coding framework was developed, based on CFIR constructs [31]. The coding framework was further discussed among authors and a coding framework including information on adoption, fidelity, acceptability, feasibility, barriers, and facilitators was developed. To compare the implementation process in each PHC, matrices were used.

## Results

### Implementation process outcomes

The pilot study took place between September 2019 and May 2020. The three PHCs included are located on the outskirts of the provincial capital, serve vulnerable rural and semi-rural populations, and are staffed by physicians, nurses, and community health workers, among others. Overall, the implementation level of the intervention was heterogeneous across PHCs and varied depending on components (see Table 2).

**Table 2:** Level of implementation of the strategy

Component	Outcome measures	Details
Training	Adopted	Implemented in all PHCs. Some professionals were not trained because they were absent or worked on other duties on the training date
Flowcharts	Partially adopted Low fidelity	Flowcharts were used in all PHCs, but risk screening was partially implemented. Indication of serology was increased without using risk assessment
Reminders	Adopted	Reminder posters were used in all centres
Frequently asked questions sheet	Not adopted	Few informants mentioned knowing or having used this component
Register of at-risk persons and positive cases	Poorly adopted Low fidelity Low feasibility	No registers of persons at risk were implemented and, registers of positive cases were used in only one PHC. Clinical records of positive patients were labeled
Management facilitators	Partially adopted Low fidelity Low feasibility	All PHCs appointed facilitators, but their performance and commitment were variable

The training component of the intervention was implemented in the three participating PHCs. Although healthcare staff positively described training, it did not reach all professionals, and there was no internal or cascade training. Health care providers who had not attended the training session did not know the intervention well enough. Participants at all sites mentioned having implemented the flowcharts, as confirmed by observations. Risk screening was only implemented partially, unsystematically, and differently from what had been proposed (low fidelity). Some respondents used the screening questions as a guide to prescribing serologic tests. In two PHCs, the Chagas serologic test was prescribed for all women of childbearing age without risk screening.

Participants thought reminders were helpful and implemented their use, especially posters. However, cards with frequently asked questions were rarely used because no treatments were carried out. Despite participants’ willingness to implement the register of positive and at-risk cases early, uptake was low. When components were implemented, the implementation did not conform to proposed indications (low fidelity). In fact, at one PHC, staff developed a register for patients receiving treatment, but it was not used because patients were not treated. In another PHC, registration of positive cases was abandoned after a few entries. All PHCs used labels to signal positive case records but not for at-risk patients. Management facilitators were appointed in the three PHCs, but their performance had mixed results. Often, facilitators did not fulfil the proposed role; indeed, many health care providers were unaware of this component. Replacement of facilitators during the study due to leave of absence or transfers affected the performance of this role.

The health care teams well received the intervention. Participants regarded the components of the intervention as acceptable and relevant but pointed out critical aspects of the feasibility of the intervention. Participants perceived that the implementation strategy had a low impact and that the main positive effect was the increased diagnostic tests. Although more tests of women of childbearing age were prescribed, the risk was not screened. This increase in tests was only seen during the first months of the intervention. Limiting factors were blood samples not sent to the central laboratory due to summer recess, work overload of the laboratory that had to process COVID-19 tests, and the interruption of healthcare services at PHCs because of the pandemic. Significantly, some participants’ unwillingness to start treatment undermined the proactive search for positive cases.

Despite the increased testing, few new cases were detected, and no treatment was started. Reasons provided by participating health providers included patients´ comorbidities, patients who were outside the recommended age range for treatment, and health providers’ reluctance to administer treatment at the first level of care. Several professionals expressed concerns, and one physician refused to indicate treatment altogether, as they thought adverse effects outweighed benefits and feared legal actions from patients and their families.

### Barriers and facilitators

The facilitating factors were the simplicity of the intervention, professionals' willingness to extend the indication of serologic tests to other populations, and staff commitment to the adoption of intervention components. Participants identified barriers to the implementation at different levels, such as internal factors, contextual factors, and individual-level attitudes of the intervention's target population (see Table 3). At the individual level, the most significant barrier was professionals' reluctance to treat Chagas at the first level of care due to fear of adverse effects. Sometimes, this refusal was associated with a lack of knowledge and negative experiences in the past, a lack of experience in treating the condition, and fear of medication supply shortages. Disagreement between healthcare staff on the need to treat Chagas at the PHC led to some professionals perceiving that the intervention was pointless.

**Table 3:** Barriers to the implementation

Dimension	Barrier	Description
**Inner setting**	Insufficient human resources	Insufficient human resources and time due to demand for care and contextual factors
	Low commitment	Lack of key actors (directors and/or facilitators) with a greater commitment to the intervention due to leave of absence or other reasons
	Insufficient knowledge about intervention	Professionals with low knowledge of the intervention
**Outer setting and context**	Lack of critical resources	Limited access to serology during much of the intervention period
	Health emergencies	Health emergencies (dengue outbreak and COVID-19 pandemic) absorbed time and resources and/or limited care activity in the centers
**Individuals involved**	Beliefs and attitudes related to the intervention	Reluctance to starting treatment at the first level of care for fear of side effects or lack of knowledge

### Inner setting

Insufficient human resources to carry out intervention activities at the PHCs was one barrier identified by professionals. The lack of human resources resulting from annual leave, the annual summer recess and other priorities, such as dengue epidemic containment and other health programs, negatively affected the capacity to implement the intervention. Informants highlighted the lack of time to cope with the demand for care.

PHC directors’ and facilitators’ low commitment disrupted the implementation. Staff turnover and frequent leaves of absence were obstacles to promoting implementation components. Limited understanding of the intervention, because some staff had not taken part in the training sessions, was a barrier to adopting the components; additionally, internal communication and training among staff did not work.

### Outer setting

External factors, such as lack of resources for sampling at the central laboratory and the COVID-19 pandemic, were critical aspects that negatively affected the implementation and continuity of the intervention. A limited sampling at the central laboratory resulted from the summer recess and the COVID-19 pandemic. During the summer months, serologic tests were only performed in emergencies, which did not include Chagas tests. Additionally, there was a shortage of reagents for other pathologies, which contributed to an interruption of sampling at PHCs. Resistencia city was a hotspot during the pandemic, and the central laboratory that processed Chagas tests was the only one in the province that processed COVID-19 tests. The pandemic and the associated social isolation measures led to a disruption of healthcare activities at PHCs. Since the pandemic, healthcare staff numbers have been reduced, and some staff has been reassigned to tasks related to pandemic control.

## Discussion

This research designed an implementation strategy to improve using Chagas guidelines focused on attending women of childbearing age and children at the primary healthcare level and piloted it. Implementing the intervention at the first level of care was uneven across PHCs. During the last months of the intervention, the COVID-19 pandemic and associated measures affected the organization of the health system, the regular operation of PHCs, and the implementation study. Some components have been fully or partially implemented, such as flowcharts and the role of the facilitator. In contrast, the implementation of other components, such as registries of positive cases and people at risk, has been low. Although the target population perceived the intervention as relevant and acceptable, it had a limited impact. Positively, according to interviewed professionals, the population indicated for serological testing increased in the first months of the intervention but then discontinued because of contextual barriers. A few new positive cases were registered during the study, and no treatment was started in the participating centers. Although the intervention proposed that women of childbearing age, and children and adolescents at risk, and all pregnant women had to be tested, the decision was based only on medical criteria.

The main factors that affected implementation were the lack of diagnostic supplies (linked to contextual barriers), the change of authorities, staff shortages, and, in last months, the health emergency caused by the COVID-19 pandemic, which interrupted the normal functioning of health care activities. The reluctance of some professionals to carry out etiological treatment of positive cases of Chagas disease at the first level of care was a considerable barrier to the adoption of the intervention.

Lessons learned to improve design and implementation are highlighted, following the dimensions of the CFIR model [32]. Concerning the characteristics of the intervention, training needs to be improved to strengthen health personnel's confidence in the quality and strength of the evidence, particularly regarding etiological treatment. In addition, training should be conducted regularly, aiming to train the entire target population and include barriers or difficulties identified in process evaluations. It is critical to work on health providers´ knowledge and perception of the disease, its impact, and treatment possibilities. Producing changes in beliefs, attitudes, and behaviors on both health personnel (medical and non-medical staff) and patients is one of the significant challenges faced when dealing with Chagas disease, as reported in the literature [15]. The reluctance of health care providers to indicate etiological treatment at the first level of care, a barrier identified in previous studies and formative research [13–15], persisted despite training.

The main lessons learned in this study were that behavioral change strategies must be implemented, including support from opinion leaders, medical experts, and local health authorities. This coincides with findings from a study in another city in Argentina, recommending the inclusion of a senior physician from a specialized Chagas organization for medical queries [33]. The role of facilitator, taken by someone external to the PHCs, should be strengthened. These points align with regional research [15], which highlighted the identification of interested health workers and the specific training on disease management. The facilitator should also be included in the planning, implementation, and evaluation phases, supported by financial and/or academic incentives.

On the other hand, rapid diagnostic tests should be provided [34] in response to the identified barrier, describing that the laboratories lacked the resources to respond to the increased demand for serology. This implementation strategy would be an optimal scenario for testing "Test&Treat" models, with innovations such as the use of two rapid diagnostic tests (2 PDTs) to confirm infection [34, 35] and new antiparasitic regimens to deliver treatments with a better safety profile – BENDITA [36]; BETTY [37], TESEO [38]; NuestroBen [39]. Alonso-Padilla et al. 2019 [15] proposed innovative, simpler and faster diagnostic strategies, LAMPs for antigens and rapid serological tests (T. cruzi-specific IgGs, rapid diagnostic tests), and new therapeutic regimens.

It is essential to actively involve the local Chagas program referents and other local authorities and serology laboratories to support and sustain the strategy. As stated by Alonso-Padilla et al. [15], based on the experience in Bolivia, a vertical-to-horizontal healthcare model transition is needed to improve access for people at risk, coordinating with local authorities to adopt the strategy and promote intersectoral policies. The Chagas program implemented in another city in Argentina also refers to the importance of the participation of local authorities in generating trust in the intervention [13]. Regarding the inner setting, supplies such as laboratory and electrocardiogram equipment and human resources should be guaranteed, as shown in previous studies [13]. It is necessary to improve networking and communication within the centers. However, the assessment of the implementation climate, the ability to make changes, and the participants' willingness is also important to incorporate this aspect into the training. Involving the National Chagas program and the maternal and child health program would be essential to strengthen networks, facilitate practices, and improve materials and supplies, necessary for timely diagnosis and medication access, which is free of charge in Argentina, as well as to guarantee sustainability.

Our study has limitations that should be acknowledged. The population studied did not include healthcare users, stakeholders, community health agents or other technical professionals working in the primary care settings analyzed. Since the main target of the implementation actions was the PHC team, we focused on their perspective. Additionally, the COVID-19 pandemic disrupted health care activities at PHCs, making it difficult to include users in the process evaluation.

## Conclusions

In this study, we designed and evaluated the feasibility of an implementation strategy to improve the adoption of national guidelines for managing Chagas disease at the primary care level. The process evaluation showed that the components of the implementation strategy were acceptable to the primary healthcare providers; however, their adoption was uneven across PHCs. Although some of the components were partial to fully adopted, the fidelity of their implementation was low. The professionals´ reluctance to administer etiological treatment at the PHCs hindered the intervention and contextual and health care center-related barriers, such as staff shortages, lack of diagnostic supplies, and COVID-19 pandemic, limited its impact. Our findings show the need to improve the implementation approach using behavioral change strategies such as support actions offered by key actors to address barriers. Strategies should be further refined to facilitate implementation of *T. cruzi* infection management into primary healthcare level, with accessible diagnosis, treatment, and follow-up of this condition.

## Supplementary Information

**Additional file 1.** Flowchart for the management of Chagas in pregnant women, Spanish version (original version). Information for gynecologists, obstetricians, midwives and general and family practitioners for the management of Chagas in pregnant women.

**Additional file 2.** Flowchart for the management of Chagas in pregnant women, English version.

**Additional file 3.** Flowchart for the management of Chagas in women of childbearing age, Spanish version (original version). Information for gynecologists, obstetricians, midwives and general and family practitioners, for the management of Chagas in women of childbearing age.

**Additional file 4.** Flowchart for the management of Chagas in women of childbearing age, English version.

**Additional file 5.** Flowchart for the management of Chagas in children and adolescents, Spanish version (original version). Information for pediatricians, gynecologists, general practitioners, and family doctors for the management of Chagas in children and adolescents.

**Additional file 6.** Flowchart for the management of Chagas in children and adolescents, English version.

**Additional file 7.** Reminder for the management of Chagas in women of childbearing age, Spanish version (original version). Poster remaining on how to manage Chagas in women of childbearing age.

**Additional file 8.** Reminder for the management of Chagas in children and adolescents, Spanish version (original version). Poster remaining on how to manage Chagas in children and adolescents.

**Additional file 9.** Reminder for Chagas, Spanish version (original version). Stickers to remind the relevance of Chagas.
Legend for Additional files 1-9: These materials were developed after extensive formative research, working with designers and researchers from different disciplines, and are the fundamental components of the implementation strategy. It was all produced by the research team based on the information and recommendations of the national Chagas guide, which is similar to Chagas guidelines from other regions of Latin America. Appropriate copyright permission to use images of the company logos was obtained.

**Additional file 10.** English language translation of the additional files 7, 8 and 9.

**Additional file 11.** Interview guide, English language version. Interview guide for the semi-structured interviews, developed by the research team.

**Additional file 12.** Completed Standards for Reporting Implementation Studies (StaRI) checklist.

## Data Availability

Data sharing is not applicable to this article as no datasets were generated or analyzed during the current study. To obtain access to the data analysed in the study, please contact Karen Klein (kklein@iecs.org.ar).

## Abbreviations

CFIR: Consolidated Framework for Implementation Research
PHCs: Primary healthcare centers
REDCap: Research Electronic Data Capture

## References

[1] Chagas disease in Latin America: an epidemiological update based on 2010 estimates. Wkly Epidemiol Rec [Internet]. 2015; (90):[33–43 pp.]. Available from http://www.who.int/wer/2015/wer9006.pdf?ua=1. 25671846

[2] Coura JR, Viñas PA (2010). Chagas disease: a new worldwide challenge. Nature.

[3] Schmunis GA, Yadon ZE (2010). Chagas disease: a Latin American health problem becoming a world health problem. Acta Trop.

[4] Spillmann C, Burrone MS, Coto H (2013). Análisis de la situación epidemiológica de la enfermedad de Chagas en Argentina: avances en el control 2012. Rev Argent Salud Publica..

[5] Sosa-Estani S (2005). Congenital transmission of Trypanosoma cruzi infection in Argentina. Rev Soc Bras Med Trop.

[6] Sosa-Estani S, Cura E, Velazquez E, Yampotis C, Segura EL (2009). Etiological treatment of young women infected with Trypanosoma cruzi, and prevention of congenital transmission. Rev Soc Bras Med Trop.

[7] Fabbro DL, Danesi E, Olivera V, Codebó MO, Denner S, Heredia C (2014). Trypanocide Treatment of Women Infected with Trypanosoma cruzi and Its effect on preventing congenital Chagas. PLoS Negl Trop Dis.

[8] Moscatelli G, Moroni S, García-Bournissen F, Ballering G, Bisio M, Freilij H (2015). Prevention of congenital Chagas through treatment of girls and women of childbearing age. Mem Inst Oswaldo Cruz.

[9] Guía para la atención al paciente infectado con Trypanosoma cruzi (Enfermedad de Chagas) Buenos Aires: Secretaría de Gobierno de Salud de la Nación; 2018. Available from https://bancos.salud.gob.ar/sites/default/files/2020-01/chagas-atencion-paciente-infectado-2018.pdf.

[10] Guidelines for the diagnosis and treatment of Chagas disease. Washington, D.C.: Pan American Health Organization; 2019 Cited Pan American Health Organization. Available from https://iris.paho.org/bitstream/handle/10665.2/49653/9789275120439_eng.pdf.

[11] Sosa-Estani S, Colantonio L, Segura EL (2012). Therapy of Chagas disease: implications for levels of prevention. J Trop Med.

[12] Sosa-Estani S, Altcheh J, Riarte A, Freilij H, Fernández M (2015). Lineamientos básicos del tratamiento etiológico de enfermedad de Chagas. Medicina (Buenos Aires)..

[13] Klein K, Burrone MS, Alonso JP, Ares LR, Martí SG, Lavenia A (2017). Estrategia para mejorar el acceso al tratamiento etiológico para la enfermedad de Chagas en el primer nivel de atención en Argentina. Rev Panam Salud Publica..

[14] Roberti J, et al. Abordaje de la enfermedad de Chagas en el primer nivel de atención: investigación cualitativa en una zona endémica de Argentina. Interface - Comunicação, Saúde, Educação [online]. 2020;24:e200104. Available from: 10.1590/interface.200104.

[15] Alonso-Padilla J, Cortés-Serra N, Pinazo MJ, Bottazzi ME, Abril M, Barreira F (2019). Strategies to enhance access to diagnosis and treatment for Chagas disease patients in Latin America. Expert Rev Anti Infect Ther.

[16] Damasceno RF, Sabino EC, Ferreira AM, Ribeiro ALP, Moreira HF, Prates TEC (2020). Challenges in the care of patients with Chagas disease in the Brazilian public health system: a qualitative study with primary health care doctors. PLoS Negl Trop Dis.

[17] Pinazo M-J, Pinto J, Ortiz L, Sánchez J, García W, Saravia R (2017). A strategy for scaling up access to comprehensive care in adults with Chagas disease in endemic countries: The Bolivian Chagas Platform. PLoS Negl Trop Dis.

[18] Althabe F, Buekens P, Bergel E, Belizan JM, Campbell MK, Moss N (2008). A behavioral intervention to improve obstetrical care. N Engl J Med.

[19] Iniciativa Medicamentos para Enfermedades Olvidadas (DNDi). Available from http://www.dndial.org/es/dndi-en-america-latina.html.

[20] Marchiol A, Forsyth C, Bernal O, Valencia Hernández C, Cucunubá Z, Pachón Abril E (2017). Increasing access to comprehensive care for Chagas disease: development of a patient-centered model in Colombia. Rev Panam Salud Publica.

[21] Alonge O, Rodriguez DC, Brandes N, Geng E, Reveiz L, Peters DH (2019). How is implementation research applied to advance health in low-income and middle-income countries?. BMJ Glob Health.

[22] Damschroder LJ, Aron DC, Keith RE, Kirsh SR, Alexander JA, Lowery JC (2009). Fostering implementation of health services research findings into practice: a consolidated framework for advancing implementation science. Implement Sci.

[23] Qualitative Research in Implementation Science. Division of Cancer Control and Population Sciences: U.S. National Institutes of Health, National Cancer Institute; 2019. Available from https://cancercontrol.cancer.gov/sites/default/files/2020-09/nci-dccps-implementationscience-whitepaper.pdf.

[24] Hamilton AB, Finley EP (2019). Qualitative methods in implementation research: an introduction. Psychiatry Res.

[25] Pinnock H, Barwick M, Carpenter CR, Eldridge S, Grandes G, Griffiths CJ (2017). Standards for Reporting Implementation Studies (StaRI) statement. BMJ.

[26] Sistema integrado de información sanitaria argentina (SIISA). Available from https://sisa.msal.gov.ar/sisa/#sisa.

[27] Chan WV, Pearson TA, Bennett GC, Cushman WC, Gaziano TA, Gorman PN (2017). ACC/AHA special report: clinical practice guideline implementation strategies: a summary of systematic reviews by the NHLBI implementation science work group: a report of the American College of Cardiology/American heart association task force on clinical practice guidelines. J Am Coll Cardiol.

[28] Marchiol A, Forsyth C, Bernal O, Valencia Hernández C, Cucunubá ZM, Pachón Abril E (2017). Increasing access to comprehensive care for Chagas disease: development of a patient-centered model in Colombia. Rev Panam Salud Publica.

[29] Proctor E, Silmere H, Raghavan R, Hovmand P, Aarons G, Bunger A (2011). Outcomes for implementation research: conceptual distinctions, measurement challenges, and research agenda. Adm Policy Ment Health.

[30] Holtrop JS, Rabin BA, Glasgow RE (2018). Qualitative approaches to use of the RE-AIM framework: rationale and methods. BMC Health Serv Res.

[31] Gibbs GR. Analyzing qualitative data. SAGE Publications, Ltd; 2007. 10.4135/9781849208574.

[32] VanDevanter N, Kumar P, Nguyen N, Nguyen L, Nguyen T, Stillman F (2017). Application of the Consolidated Framework for Implementation research to assess factors that may influence implementation of tobacco use treatment guidelines in the Viet Nam public health care delivery system. Implement Sci.

[33] Pereiro AC, Gold S (2019). Building an innovative Chagas disease program for primary care units, in an urban non- endemic city. BMC Public Health.

[34] Lopez-Albizu C, Danesi E, Piorno P, Fernandez M, García Campos F, Scollo K (2020). Rapid diagnostic tests for trypanosoma cruzi infection: field evaluation of two registered kits in a region of endemicity and a region of nonendemicity in Argentina. J Clin Microbiol..

[35] Lozano D, Rojas L, Méndez S, Casellas A, Sanz S, Ortiz L (2019). Use of rapid diagnostic tests (RDTs) for conclusive diagnosis of chronic Chagas disease – field implementation in the Bolivian Chaco region. PLoS Negl Trop Dis.

[36] Torrico F, Gascón J, Barreira F, Blum B, Almeida IC, Alonso-Vega C (2021). New regimens of benznidazole monotherapy and in combination with fosravuconazole for treatment of Chagas disease (BENDITA): a phase 2, double-blind, randomised trial. Lancet Infect Dis.

[37] Cafferata ML, Toscani MA, Althabe F, Belizán JM, Bergel E, Berrueta M (2020). Short-course Benznidazole treatment to reduce Trypanosoma cruzi parasitic load in women of reproductive age (BETTY): a non-inferiority randomized controlled trial study protocol. Reprod Health.

[38] New Therapies and Biomarkers for Chagas Infection (TESEO). ClinicalTrials.gov Identifier: NCT03981523 U.S.: National Library of Medicine 2019. Available from https://clinicaltrials.gov/ct2/show/NCT03981523.

[39] New Scheme for Treatment With Benznidazole (NuestroBen). ClinicalTrials.gov Identifier: NCT04897516 U.S.: National Library of Medicine 2021. Available from https://clinicaltrials.gov/ct2/show/NCT04897516?term=NuestroBen&draw=2&rank=1.

