# Supplementary material for: Design and feasibility of an implementation strategy to address Chagas guidelines engagement focused on attending women of childbearing age and children at the primary healthcare level in Argentina: a pilot study
Source: BMC Prim Care. 2022 Nov 8;23:277. doi: 10.1186/s12875-022-01886-6 (PMC9643922; doi:10.1186/s12875-022-01886-6)
Supplement: Supplementary file 1 — Additional file 1. Flowchart for the management of Chagas in pregnant women, Spanish version (original version). Information for gynecologists, obstetricians, midwives and general and family practitioners for the management of Chagas in pregnant women. [file 12875_2022_1886_MOESM1_ESM.pdf]

ABORDAJE DEL CHAGAS EN EMBARAZADAS

Información para ginecólogos, obstetras, obstétricas y médicos generalistas y de familia.

MUJERES EMBARAZADAS

"Si detectamos que vos tenés Chagas, tu bebé también podría tenerlo. Es importante que conozcas tu diagnóstico porque se puede curar haciendo un tratamiento."

Hacer el estudio de sangre para el diagnóstico.

Hacer la orden correspondiente: **Serología por dos técnicas.**

HACER SEGUIMIENTO DEL RESULTADO

RESULTADO DISCORDANTE
1 prueba negativa y 1 positiva

Realizar una prueba serológica más (para confirmar el diagnóstico)

RESULTADO NEGATIVO
2 pruebas negativas

"Contale a tu familia y amigas que te hiciste este estudio. Es importante que todas las mujeres que pueden quedar embarazadas sepan si tienen Chagas"

RESULTADO POSITIVO
2 pruebas positivas

Gestionar el contacto con hermanos e hijos de la paciente.

Recordarle que es importantes saber si el bebé es positivo o negativo.(*) Si el bebé tiene Chagas, explicarle que debe recibir un tratamiento.

CONTARLE A LA MUJER QUE ELLA TAMBIÉN DEBERÍA HACER EL TRATAMIENTO UNA VEZ FINALIZADO EL EMBARAZO

NOTIFICAR el caso.

MARCAR la historia clínica de alguna forma que resulte identificable para el personal de salud la positividad para Chagas.

REGISTRAR en la historia clínica el resultado positivo para CHAGAS para ALERTAR al personal de salud de la importancia del seguimiento posterior de la madre y el bebé

(*) Diagnóstico al nacimiento o a los 10 meses si dio negativo al nacer.

IECS INSTITUTO DE EFECTIVIDAD CLÍNICA Y SANITARIA
