# Supplementary material for: Design and feasibility of an implementation strategy to address Chagas guidelines engagement focused on attending women of childbearing age and children at the primary healthcare level in Argentina: a pilot study
Source: BMC Prim Care. 2022 Nov 8;23:277. doi: 10.1186/s12875-022-01886-6 (PMC9643922; doi:10.1186/s12875-022-01886-6)
Supplement: Supplementary file 2 — Additional file 2. Flowchart for the management of Chagas in pregnant women, English version. [file 12875_2022_1886_MOESM2_ESM.pdf]

MANAGEMENT OF CHAGAS DISEASE IN PREGNANT WOMEN

Information for gynecologists, obstetricians, midwives and general and family practitioners.

PREGNANT WOMEN

If we detect that you have Chagas disease, your baby could also have it. It is important that you know the diagnosis because it can be cured with treatment.

Blood test for diagnosis.

Make the corresponding medical order: **Serology by two techniques.**

TRACK THE RESULT

DISCORDANT RESULT
1 negative and 1 positive test

Perform an additional serological test (to confirm the diagnosis)

NEGATIVE RESULT
2 negative tests

"Tell your family and friends that you had this test. It is important that all women who may become pregnant know if they have Chagas disease."

POSITIVE RESULT
2 positive tests

Manage contact with siblings and children of the patient.

Remind her that it is important to to know if the baby is positive or negative (*). If the baby has Chagas disease, explain that he/she must be treated.

(*) Diagnosis at birth, or at 10 months of age if negative at birth.

TELL THE WOMAN THAT SHE SHOULD ALSO UNDERGO THE TREATMENT AFTER THE PREGNANCY IS OVER

NOTIFY THE CASE

MARK the medical record in a way that makes Chagas positivity identifiable to health personnel.

REGISTER the positive result for CHAGAS in the medical record to ALERT health personnel of the importance of subsequent follow-up of the mother and baby.
