# Supplementary material for: Design and feasibility of an implementation strategy to address Chagas guidelines engagement focused on attending women of childbearing age and children at the primary healthcare level in Argentina: a pilot study
Source: BMC Prim Care. 2022 Nov 8;23:277. doi: 10.1186/s12875-022-01886-6 (PMC9643922; doi:10.1186/s12875-022-01886-6)
Supplement: Supplementary file 3 — Additional file 3. Flowchart for the management of Chagas in women of childbearing age, Spanish version (original version). Information for gynecologists, obstetricians, midwives and general and family practitioners, for the management of Chagas in women of childbearing age. [file 12875_2022_1886_MOESM3_ESM.pdf]

CÓMO DIAGNOSTICAR Y TRATAR EL CHAGAS

Información para ginecólogos, obstetras, obstétricas y médicos generalistas y de familia.

MUJERES EN EDAD FÉRTIL

HACER LAS SIGUIENTES PREGUNTAS:

1. ¿Alguna vez te hiciste un análisis de Chagas y te dio positivo?
2. ¿Sabés si tu mamá, hermano/a o tus hijos tuvieron o tienen Chagas?
3. ¿Conocés la vinchuca? ¿Encontraste o tuviste alguna vez vinchucas en tu casa?
4. ¿Tu casa tiene algunas de estas características?:
   - Techo de paja, palma o varillas
   - Paredes con grietas, huecos o no revocadas.
   - Depósitos, corrales o Gallineros anexos a la casa
   - Animales en el interior.
5. ¿Recibiste alguna vez una transfusión de sangre o un trasplante de órganos?
6. ¿Naciste, viviste o viajaste a zonas rurales endémicas?

   (Chaco, Formosa, Santiago del Estero, San Juan, Catamarca, Mendoza, Córdoba, La Rioja, Salta, Bolivia o Brasil)

SI LA PACIENTE **RESPONDE SÍ** A UNA O MÁS DE ESTAS PREGUNTAS, TIENE RIESGO DE ESTAR INFECTADO

NO

SI

HISTORIA CLÍNICA

Anotar que la paciente tiene riesgo de Chagas

“Si diagnosticamos que tenés Chagas, te podés tratar y podés evitar transmitirle la enfermedad a futuros hijos”.

1. Explicarle por qué es importante saber si está infectada.
2. Proponerle hacerse un estudio de sangre

Hacer la orden correspondiente: **Serología por dos técnicas.**

HACER SEGUIMIENTO DEL RESULTADO

RESULTADO DISCORDANTE
1 prueba negativa y 1 positiva

Realizar una prueba serológica más (para confirmar el diagnótico)

RESULTADO NEGATIVO
2 pruebas negativas

“Contale a tu familia y amigas que te hiciste este estudio. Es importante que todas las mujeres que pueden quedar embarazadas sepan si tienen Chagas”

RESULTADO POSITIVO
2 pruebas positivas

Hablar con la paciente y explicarle que sus hermanos e hijos también se tienen que realizar el análisis de sangre.

NOTIFICAR
el caso.

MARCAR
la historia clínica de alguna forma que resulte identificable para el personal de salud la positividad para Chagas.

REGISTRAR
en la historia clínica el resultado y la información de tratamiento y seguimiento.

Control clínico pre-tratamiento

Exámen físico completo, electrocardiograma, ecocardiograma radiografía de tórax y laboratorio (hemograma, eritrosedimentación, creatinina o urea, hepatograma; y prueba de embarazo).

Verificar ausencia de:
embarazo, insuficiencia cardíaca avanzada, insuficiencia renal, insuficiencia hepática, o trastornos neurológicos graves.

GARANTIZAR AUSENCIA DE VINCHUCAS EN EL DOMICILIO (*)

GARANTIZAR ANTICONCEPCIÓN

REALIZAR TRATAMIENTO ETIOLÓGICO

DÍA 1 — **BENZNIDAZOL O NIFURTIMOX** durante 60 días luego de las comidas (1)

(Leer Guía para la atención al paciente infectado con Trypanosoma cruzi -Enfermedad de Chagas- 2018)

DÍA 7 — Seguimiento de eventos adversos (EA)

DÍAS 15 / 20 — Nuevo seguimiento de EA y solicitar estudios de creatinina o urea, hemograma y transaminasas

DÍAS 30, 45 Y 60 — Últimos seguimientos de EA.

(1) **Benznidazol** : 5-10 MG/KG/DÍA Administrados en dos tomas diarias (cada 12 horas) luego de las comidas. Se recomienda dosis máxima de 300 mg/día

Nifurtimox: • Personas con menos de 40 Kg*. 10 a 20 mg /Kd/dia en tres tomas diarias (cada 8 horas). • Adolescentes** entre 40 y 60 Kg, 12,5 a 15 mg/Kg/dia

• Personas con más de 60 Kg, 8 a 10 mg/Kg/dia en tres tomas diarias (cada 8 horas). Con una dosis máxima de 720 mg/dia.

(*) DAR AVISO AL PROGRAMA PROVINCIAL DE CHAGAS PREVIO AL TRATAMIENTO PARA REALIZAR EL CONTROL VECTORIAL EN LA CASA.

IECS
INSTITUTO DE EFECTIVIDAD CLÍNICA Y SANITARIA
