# Supplementary material for: Design and feasibility of an implementation strategy to address Chagas guidelines engagement focused on attending women of childbearing age and children at the primary healthcare level in Argentina: a pilot study
Source: BMC Prim Care. 2022 Nov 8;23:277. doi: 10.1186/s12875-022-01886-6 (PMC9643922; doi:10.1186/s12875-022-01886-6)
Supplement: Supplementary file 4 — Additional file 4. Flowchart for the management of Chagas in women of childbearing age, English version. [file 12875_2022_1886_MOESM4_ESM.pdf]

HOW TO DIAGNOSE AND TREAT CHAGAS DISEASE

Information for gynecologists, obstetricians, midwives, and general and family practitioners.

WOMEN UN REPRODUCTIVE AGE → **MAKE THE FOLOWING QUESTIONS:**

1. Have you ever been tested positive for Chagas disease?
2. Do you know if your mom, sibling or children had or have Chagas?
3. Do you know the vinchuca? Have you ever found or had vinchucas in your home?
4. Does your house have any of these characteristics?:
   - Roof made of thatch, palm or rods
   - Walls with cracks, holes or not plastered.
   - Deposits, corrals or chicken coops attached to the house.
   - Animals inside.
5. Have you ever received a blood transfusion or organ transplant?
6. Were you born in, lived in or traveled to rural endemic areas?

   (Chaco, Formosa, Santiago del Estero, San Juan, Catamarca, Mendoza, Córdoba, La Rioja, Salta, Bolivia o Brasil)

IF THE PATIENT **ANSWERS YES** TO ONE OR MORE OF THESE QUESTIONS, SHE IS AT RISK OF BE INFECTED

NO

YES → **MEDICAL RECORD** Report that the patient is at risk for Chagas infection

If we diagnose you with Chagas infection, you can be treated and avoid transmitting the infection to future children.

1. Explain why it is important to know if she is infected.
2. Propose to her to have a blood test

Make the corresponding medical order: **Serology** by two techniques.

TRACK THE RESULT

DISCORDANT RESULT
1 negative and 1 positive test

Perform an additional serological test (to confirm the diagnosis)

NEGATIVE RESULT
2 negative tests

"Tell your family and friends that you had this test. It is important that all women who may become pregnant know if they have Chagas disease."

POSITIVE RESULT
2 positive tests

Talk to the patient and explain that her siblings and children also have to be blood tested.

NOTIFY THE CASE

MARK the medical record in a way that makes Chagas positivity identifiable to health personnel.

REGISTER in the medical record the result, and treatment and follow-up information.

Pre-treatment clinical control

Complete physical examination, electrocardiogram, echocardiogram, chest x-ray and laboratory (hemogram, erythrocyte sedimentation, creatinine or urea, hepatogram; and pregnancy test).

Verify absence of: pregnancy, advanced heart failure, renal failure, hepatic failure, or severe neurological disorders.

GUARANTEE THE ABSENCE OF VINCHUCAS AT HOME (*)

GUARANTEE CONTRACEPTION

PERFORM ETHIOLOGICAL TREATMENT

DAY 1 — **BENZNIDAZOL OR NIFURTIMOX** for 60 days after meals (1)
(Read: Guidelines for the care of patients infected with Trypanosoma cruzi -Chagas disease- 2018)

DAY 7 — Follow-up pf adverse events

DAYS 15 / 20 — New follow-up of AE and request creatinine or urea, hemogram and transaminase studies.

DAYS, 45 / 60 — Latest EA follow-ups.

(1) **Benznidazol** : 5-10 MG/KG/DAY Administered in two daily intakes (every 12 hours) after meals. Maximum dose of 300 mg/day is recommended
Nifurtimox: • Persons under 40 kg*. 10 to 20 mg /Kd/day in three daily intakes (every 8 hours). • Adolescents** between 40 and 60 kg, 12.5 to 15 mg/kg/day.
• People over 60 Kg, 8 to 10 mg/Kg/day in three daily intakes (every 8 hours). With a maximum dose of 720 mg/day.

(*) NOTIFY THE PROVINCIAL CHAGAS PROGRAM PRIOR TO TREATMENT TO CARRY OUT VECTOR CONTROL IN THE HOME

TRANSLATED WITH WWW.DEEPL.COM/TRANSLATOR (FREE VERSION)

IECS INSTITUTO DE EFECTIVIDAD CLÍNICA Y SANITARIA
