# Supplementary material for: Design and feasibility of an implementation strategy to address Chagas guidelines engagement focused on attending women of childbearing age and children at the primary healthcare level in Argentina: a pilot study
Source: BMC Prim Care. 2022 Nov 8;23:277. doi: 10.1186/s12875-022-01886-6 (PMC9643922; doi:10.1186/s12875-022-01886-6)
Supplement: Supplementary file 5 — Additional file 5. Flowchart for the management of Chagas in children and adolescents, Spanish version (original version). Information for pediatricians, gynecologists, general practitioners, and family doctors for the management of Chagas in children and adolescents. [file 12875_2022_1886_MOESM5_ESM.pdf]

CÓMO DIAGNOSTICAR Y TRATAR EL CHAGAS

Información para pediatras, ginecólogos, médicos generalistas y de familia.

NIÑOS, NIÑAS Y ADOLESCENTES

HACER LAS SIGUIENTES PREGUNTAS AL ADULTO QUE ACOMPAÑA AL PACIENTE:

1. ¿Alguna vez te hiciste un análisis de Chagas y te dio positivo?
2. ¿Sabés si tu mamá, hermano/a o tus hijos tuvieron o tienen Chagas?
3. ¿Conocés la vinchuca? ¿Encontraste o tuviste alguna vez vinchucas en tu casa?
4. ¿Tu casa tiene algunas de estas características?:
   - Techo de paja, palma o varillas
   - Paredes con grietas, huecos o no revocadas.
   - Depósitos, corrales o Gallineros anexos a la casa
   - Animales en el interior.
5. ¿Recibiste alguna vez una transfusión de sangre o un trasplante de órganos?
6. ¿El paciente nació, vivió o viajó a zonas rurales endémicas?

   (Chaco, Formosa, Santiago del Estero, San Juan, Catamarca, Mendoza, Córdoba, La Rioja, Salta, Bolivia o Brasil)

SI EL ACOMPAÑANTE O EL PACIENTE **RESPONDE SÍ** A UNA O MÁS DE ESTAS PREGUNTAS, TIENE RIESGO DE ESTAR INFECTADO

NO

SI

HISTORIA CLÍNICA
Anotar que el paciente tiene riesgo de Chagas

1. Explicar al acompañante, o al paciente si corresponde, por qué es importante saber si está infectado
2. Proponerle hacerse un estudio de sangre

Hacer la orden correspondiente: **Serología por dos técnicas.**

HACER SEGUIMIENTO DEL RESULTADO

RESULTADO DISCORDANTE
1 prueba negativa y 1 positiva

Realizar una prueba serológica más (para confirmar el diagnótico)

RESULTADO NEGATIVO
2 pruebas negativas

"Contale a tu familia que el niño/la niña/el adolescente se hizo el estudio. Es importante que todos ellos sepan que también pueden tener la enfermedad."

RESULTADO POSITIVO
2 pruebas positivas

La mamá y los hermanos del paciente deben realizarse análisis de sangre para saber si están infectados.

NOTIFICAR
el caso.

MARCAR
la historia clínica de alguna forma que resulte identificable para el personal de salud la positividad para Chagas.

REGISTRAR
en la historia clínica el resultado y la información de tratamiento y seguimiento.

Control clínico pre-tratamiento

Exámen físico completo, electrocardiograma, radiografía de tórax y laboratorio (hemograma, eritrosedimentación, creatinina o urea, hepatograma)

Verificar ausencia de: insuficiencia cardíaca avanzada, insuficiencia renal, insuficiencia hepática, o trastornos neurológicos graves

GARANTIZAR AUSENCIA DE VINCHUCAS EN EL DOMICILIO (*)

REALIZAR TRATAMIENTO ETIOLÓGICO

DÍA 1 — **BENZNIDAZOL O NIFURTIMOX** durante 60 días luego de las comidas (1)
(Leer Guía para la atención al paciente infectado con Trypanosoma cruzi -Enfermedad de Chagas- 2018)

DÍA 7 — Seguimiento de eventos adversos (EA)

DÍAS 15 / 20 — Nuevo seguimiento de EA y solicitar estudios de creatinina o urea, hemograma y transaminasas

DÍAS 30, 45 Y 60 — Últimos seguimientos de EA.

(1) **Benznidazol** : 5-10 MG/KG/DÍA Administrados en dos tomas diarias (cada 12 horas) luego de las comidas. Se recomienda dosis máxima de 300 mg/día
Nifurtimox: • Personas con menos de 40 Kg*. 10 a 20 mg /Kd/dia en tres tomas diarias (cada 8 horas). • Adolescentes** entre 40 y 60 Kg, 12,5 a 15 mg/Kg/dia
• Personas con más de 60 Kg, 8 a 10 mg/Kg/dia en tres tomas diarias (cada 8 horas). Con una dosis máxima de 720 mg/dia.

(*) DAR AVISO AL PROGRAMA PROVINCIAL DE CHAGAS PREVIO AL TRATAMIENTO PARA REALIZAR EL CONTROL VECTORIAL EN LA CASA.

IECS
INSTITUTO DE EFECTIVIDAD CLÍNICA Y SANITARIA
