# Supplementary material for: Design and feasibility of an implementation strategy to address Chagas guidelines engagement focused on attending women of childbearing age and children at the primary healthcare level in Argentina: a pilot study
Source: BMC Prim Care. 2022 Nov 8;23:277. doi: 10.1186/s12875-022-01886-6 (PMC9643922; doi:10.1186/s12875-022-01886-6)
Supplement: Supplementary file 8 — Additional file 8. Reminder for the management of Chagas in children and adolescents, Spanish version (original version). Poster remaining on how to manage Chagas in children and adolescents. [file 12875_2022_1886_MOESM8_ESM.pdf]

CHAGAS

¿ATENDÉS A NIÑOS, NIÑAS Y/O ADOLESCENTES?

RECORDÁ:

HACER LAS PREGUNTAS

PARA VERIFICAR
RIESGO DE CHAGAS

SI TIENEN RIESGO DE CHAGAS

- OFRECÉ DIAGNÓSTICO
- HACÉ SEGUIMIENTO DEL RESULTADO
- TRATÁ SI CORRESPONDE

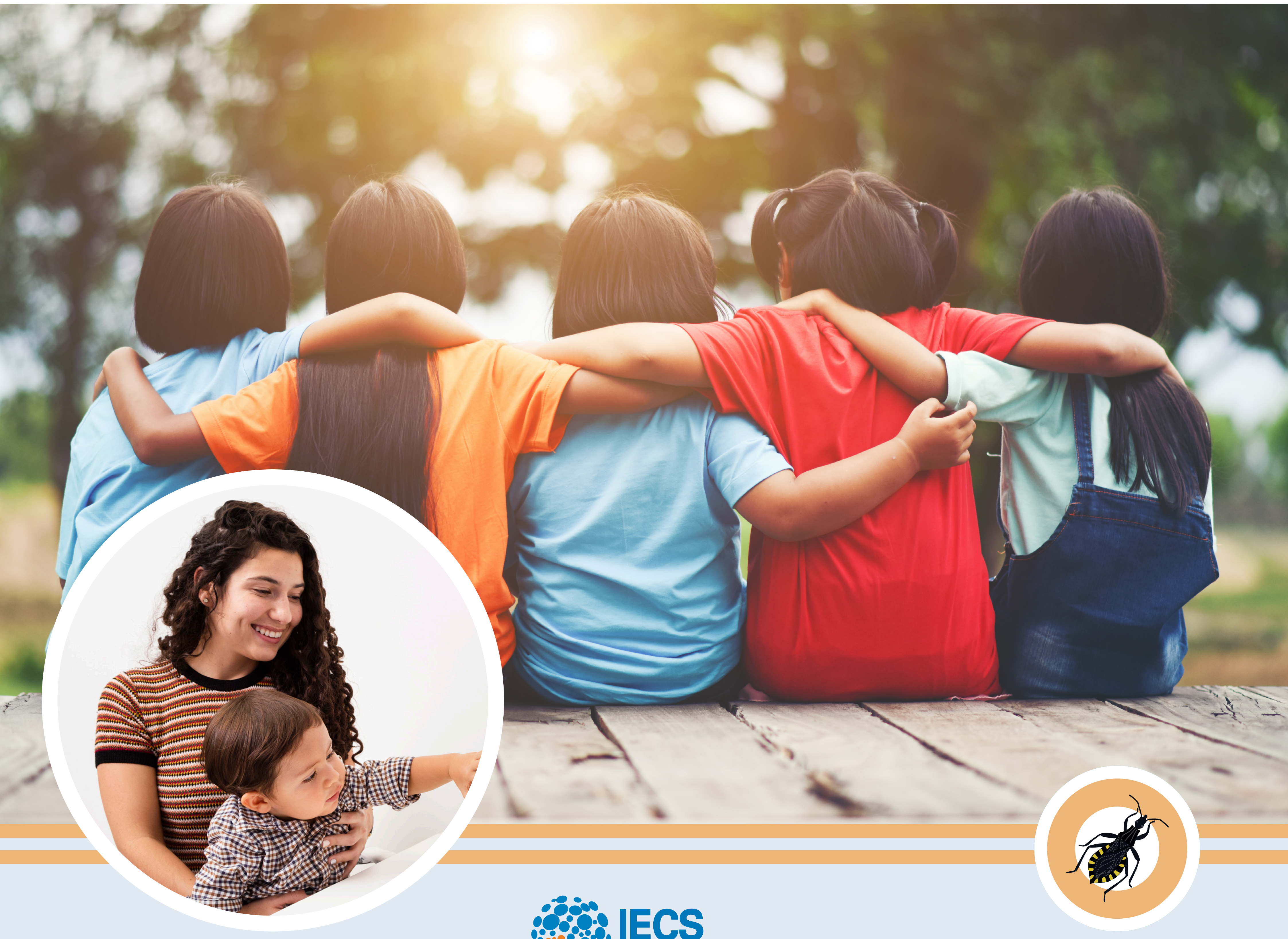

IECS
INSTITUTO DE EFECTIVIDAD CLINICA Y SANITARIA
www.iecs.org.ar
