# Supplementary figures and images for: Design and feasibility of an implementation strategy to address Chagas guidelines engagement focused on attending women of childbearing age and children at the primary healthcare level in Argentina: a pilot study
Source: BMC Prim Care. 2022 Nov 8;23:277. doi: 10.1186/s12875-022-01886-6 (PMC9643922; doi:10.1186/s12875-022-01886-6)

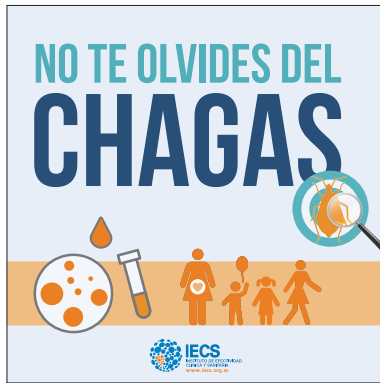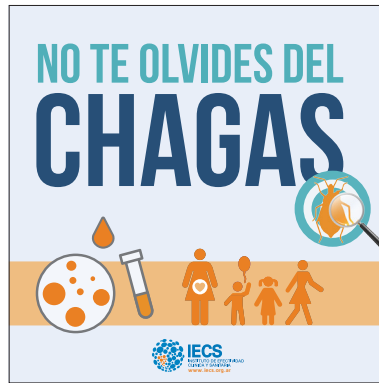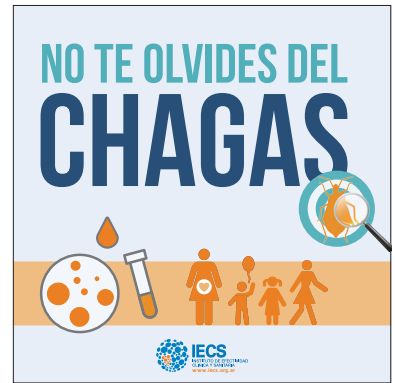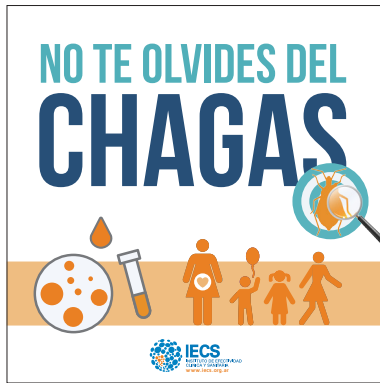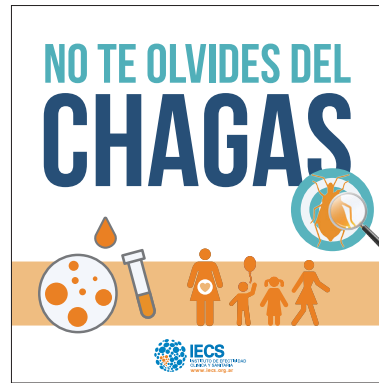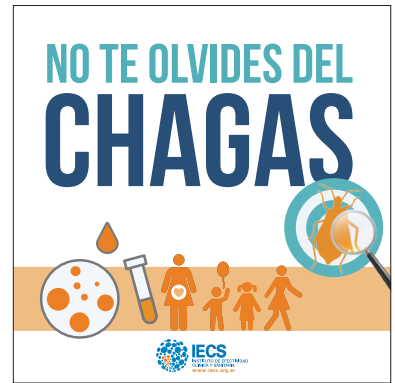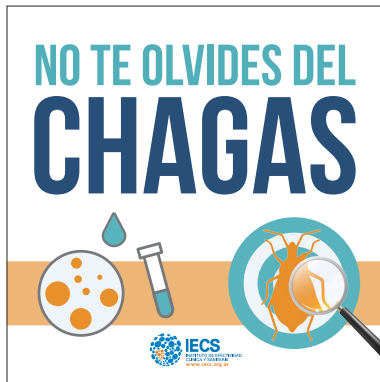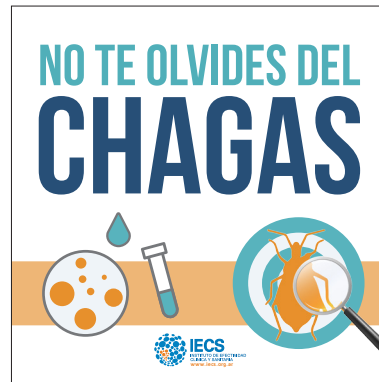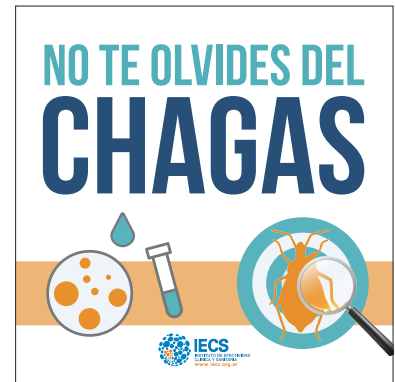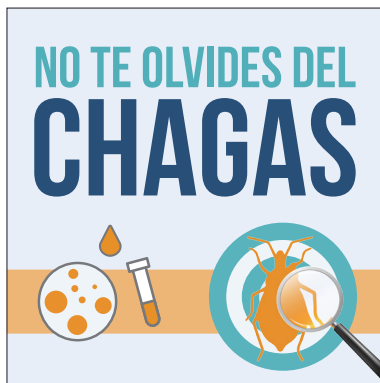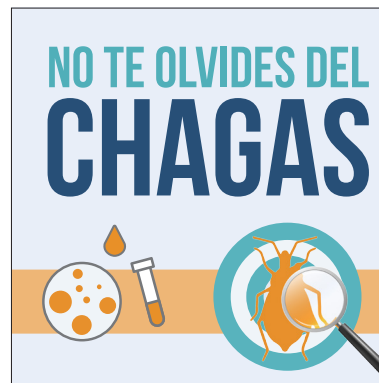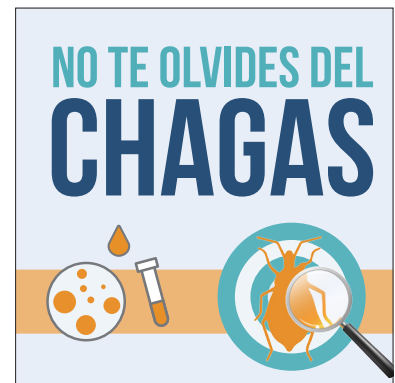

Supplement: Supplementary file 9 — Additional file 9. Reminder for Chagas, Spanish version (original version). Stickers to remind the relevance of Chagas. Legend for Additional files 1-9: These materials were developed after extensive formative research, working with designers and researchers from different disciplines, and are the fundamental components of the implementation strategy. It was all produced by the research team based on the information and recommendations of the national Chagas guide, which is similar to Chagas guidelines from other regions of Latin America. Appropriate copyright permission to use images of the company logos was obtained. [file 12875_2022_1886_MOESM9_ESM.pdf]
