# Supplementary material for: Design and feasibility of an implementation strategy to address Chagas guidelines engagement focused on attending women of childbearing age and children at the primary healthcare level in Argentina: a pilot study
Source: BMC Prim Care. 2022 Nov 8;23:277. doi: 10.1186/s12875-022-01886-6 (PMC9643922; doi:10.1186/s12875-022-01886-6)
Supplement: Supplementary file 10 — Additional file 10. English language translation of the additional files 7, 8 and 9. [file 12875_2022_1886_MOESM10_ESM.docx]

**Additional File 10**

English language translation of the additional files 7, 8 and 9.

**Additional File 7**

Do you serve women of reproductive age?

Remember:

- Make the questions to verify Chagas infection risk
- If she is at risk, offers diagnosis, follow up the result, treat if applicable

**Additional File 8**

Do you serve children and/or adolescents?

Remember:

- Make the questions to verify Chagas infection risk
- If she is at risk, offers diagnosis, follow up the result, treat if applicable

**Additional File 9**

Don't forget Chagas disease
