# Supplementary material for: Design and feasibility of an implementation strategy to address Chagas guidelines engagement focused on attending women of childbearing age and children at the primary healthcare level in Argentina: a pilot study
Source: BMC Prim Care. 2022 Nov 8;23:277. doi: 10.1186/s12875-022-01886-6 (PMC9643922; doi:10.1186/s12875-022-01886-6)
Supplement: Supplementary file 11 — Additional file 11. Interview guide, English language version. Interview guide for the semi-structured interviews, developed by the research team. [file 12875_2022_1886_MOESM11_ESM.docx]

**Additional file 11 - Interview guide**

1. What is your background?

2. What is your position and what activities do you perform at the healthcare centre?

3. What has been your role in the implementation of the intervention at this centre?

3. What intervention components have you implemented? Please tell me about the process of implementing the following components and how they have been used at the facility:

- Flowcharts/Reminders and posters/Risk screening

- Facilitators/Frequently Asked Questions sheet

- Medical records/Case registration

4. Which components have you found most useful? Which ones would have been useful if implemented? Which components were not useful or less important?

5. What do you think were the main barriers to implementing the different components?

6. How did the COVID-19 pandemic impact health care at this facility, and how did it affect the implementation of the intervention?

7. Do you think the intervention has had any impact? In which aspects?

8. What should be changed about the intervention to make it more feasible to implement or to sustain the changes over time?

9. Finally, considering all the implementation, would you add or remove any components? Why?
